# The Usefulness of Standardized Uptake Value in Differentiation between Benign and Malignant Thyroid Lesions Detected Incidentally in ^18^F-FDG PET/CT Examination

**DOI:** 10.1371/journal.pone.0109612

**Published:** 2014-10-08

**Authors:** Adam Stangierski, Kosma Woliński, Rafał Czepczyński, Agata Czarnywojtek, Martha Lodyga, Anna Wyszomirska, Małgorzata Janicka-Jedyńska, Maciej Bączyk, Marek Ruchała

**Affiliations:** 1 Department of Endocrinology, Metabolism and Internal Medicine, Poznan University of Medical Sciences, Poznań, Poland; 2 Department of Clinical Pathomorphology, Poznan University of Medical Sciences, Poznan, Poland; Memorial Sloan-Kettering Cancer Center, United States of America

## Abstract

**Introduction:**

In the last decade, (18)F-fluorodeoxyglucose (^18^F-FDG) positron emission tomography (PET and PET/CT) has become one of the major diagnostic tools used in oncology. A significant number of patients who undergo this procedure, due to non-thyroidal reasons, present incidental uptake of (^18^F-FDG) in the thyroid. The aim of the study was to compare the SUV_max_ (standardized uptake value) of thyroid focal lesions, which were incidentally found on PET/CT, in relation to the results of thyroid fine-needle aspiration biopsy (FNAB) and/or histopathological evaluation.

**Materials and Methods:**

Patients referred for PET/CT examination, due to non-thyroidal illness, presented focal ^18^F-FDG uptake in the thyroid and were advised to undergo ultrasonography (US), hormonal evaluation, FNAB and/or total thyroidectomy at our institution.

**Results:**

6614 PET/CT examinations performed in 5520 patients were analyzed. Of the 122 patients with focal thyroid ^18^F-FDG activity, 82 patients (67.2%) underwent further thyroid evaluation using FNAB. Benign lesions were diagnosed in 46 patients, malignant - in 19 patients (confirmed by post-surgical histopathology), while 17 patients had inconclusive results of cytological assessment. Mean SUV_max_ of benign lesions was 3.2±2.8 (median = 2.4), while the mean SUV_max_ value for malignant lesions was 7.1±8.2 (median = 3.5). The risk of malignancy was 16.7% for lesions with a SUV_max_ under 3, 43.8% for lesions with a SUV_max_ between 3 and 6, and 54.6% for lesions with a SUV_max_ over 6. In the group of malignant lesions, a positive correlation between the lesion’s diameter and SUV_max_ was observed (p = 0.03, r = 0.57).

**Conclusions:**

Subjects with incidental focal uptake of ^18^F-FDG in thyroid are at a high risk of thyroid malignancy. A high value of SUV_max_ further increases the risk of malignancy, indicating the necessity for further cytological or histological evaluation. However, as SUV_max_ correlated with the diameter of malignant lesions, small lesions with focal uptake of ^18^F-FDG should be interpreted cautiously.

## Introduction

In recent years, the use of (18)F-fluorodeoxyglucose (^18^F-FDG) positron emission tomography (PET and PET/CT) has evolved to become one of the fundamental diagnostic tools in the evaluation of patients with different malignancies [1], [2], [3], [4]. According to several analyses performed in the last decade, about 1.2–4.3% of subjects undergoing this procedure, due to oncological indications, present focal uptake of ^18^F-FDG in the thyroid gland [5], [6], [7], [8], [9], [10]. The estimated incidence of thyroid malignancies among these patients, confirmed with FNAB and post- surgical histopathology examination, reaches 14–50% [5], [6], [8], [9], [11], [12], [13]. The general occurrence of thyroid cancer in the total population with thyroid nodules is significantly lower, and is estimated to be less than 10% [14]. It seems, that the risk of malignancy in thyroid incidentalomas detected by ^18^F-FDG PET may be even ten times higher. Maximal standardized uptake value (SUV_max_) is used in PET examination to describe the glucose metabolism intensity in the analyzed body region, and in most cases, its higher values characterize malignant lesions [4], [15]. The aim of the current study was to compare the SUV_max_ of thyroid focal lesions found incidentally on PET/CT, performed due to oncological indications, in relation to the results of thyroid FNAB and/or histopathological evaluation.

## Materials and Methods

### Subjects

The study was performed in a single clinical center between the years of 2010 and 2013. Patients referred for PET/CT, due to non-thyroidal illness, presented focal ^18^F-FDG uptake in the thyroid and were advised to undergo endocrine evaluation including ultrasonography (US), fine needle aspiration biopsy (FNAB) and hormonal evaluation (TSH and free thyroxin concentrations) at our institution. All subjects with positive results of cytological evaluation (malignant lesion or suspicion of malignancy) were further referred for total thyroidectomy and post-operative thyroid specimens were analyzed by a pathologist. The following patient data was recorded: age, gender, basic diagnosis, size and SUV_max_ of the lesion. The Poznan University of Medical Sciences Ethical Committee in Poznan, Poland approved this study and all participating subjects provided an informed written consent.

### ^18^F-FDG PET/CT imaging

**Figure 1:**
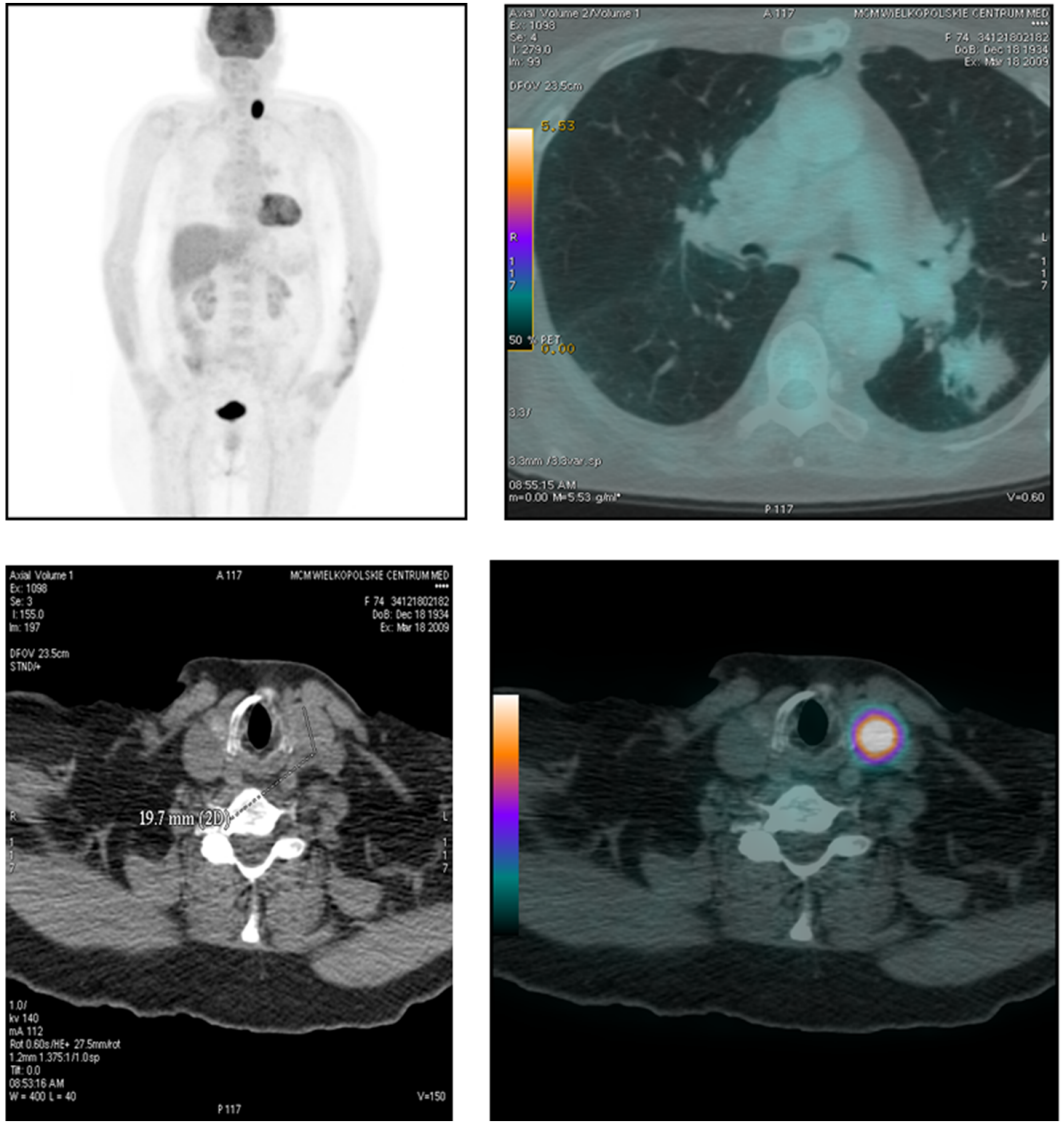
PET/CT scan performed in a female patient (74 years old) with a pulmonary tumor of indeterminant character. Incidental focal 18FDG uptake revealed in the left lobe of the thyroid gland (SUV_max_ 1.5). Colloid nodule in post-FNAB cytological evaluation.

**Figure 2:**
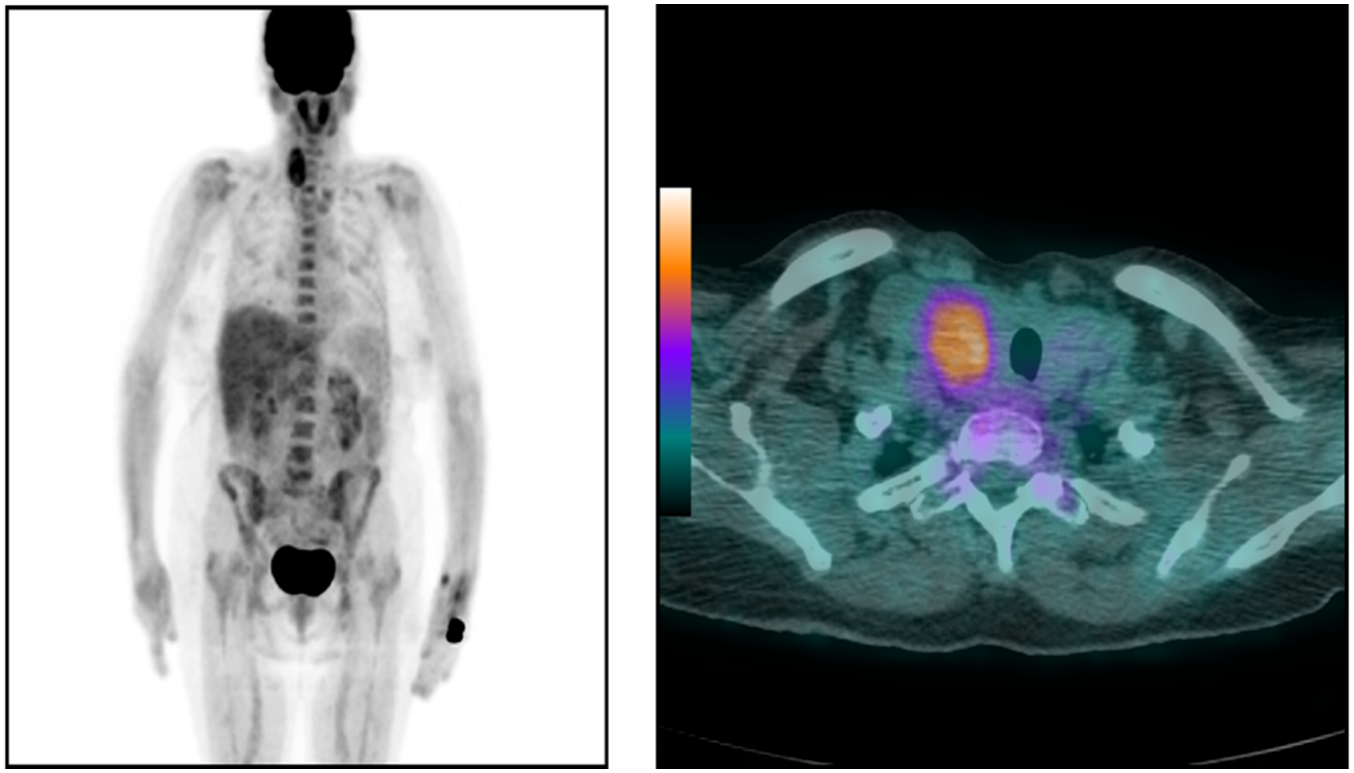
PET/CT scan of female patient (65 years old) with suspicion of colonic adenocarcinoma reoccurrence. Incidental focal 18FDG uptake revealed in the right lobe of thyroid gland (SUV_max_ 7.1). Malignancy in post-FNAB cytological assessment, further confirmed in histopathological evaluation (papillary thyroid carcinoma).

^18^F-FDG PET/CT was performed in fasting conditions using a 16-slice PET/CT scanner (Discovery ST; GE Healthcare, Milwaukee, WI). ^18^F-FDG (5 MBq per kg of body weight) was administered intravenously 60–80 min before initiation of the scan. A low-dose CT scan (140 kV, 80 mA) was performed for attenuation correction and co-registration. PET images were obtained from the base of the skull to half of the thigh with acquisition time 3 min per bed position. Acquired images were viewed by two experienced doctors: one specialized in nuclear medicine and one in radiology. Any focal uptake in the thyroid that was higher than in the adjacent structures were described as thyroid incidentaloma. The examples of focal uptake of FDG in PET/CT are presented in figures 1 and 2.

### Thyroid ultrasonography (US) and fine-needle aspiration biopsy (FNAB)

Conventional thyroid ultrasonography examinations were performed by experienced sonographers and endocrinologists in our institution’s outpatient clinic with the use of the AIXPLORER system by Supersonic Imagine and the ALOKA alpha-7 with 2–10 MHz linear transducers. Selection of lesions for further FNAB was determined by the PET/CT scan results. Fine needle aspiration biopsy was performed with G×1,5″ (0.5×40 mm) needles under ultrasound guidance. Specimens were then smeared, fixed in alcohol and further assessed by pathologists as a routine medical procedure. Final results were reported using the conventional Bethesda system [16].

### Histopathology

The final diagnosis of lesions with a suspicion for malignancy or the diagnosis of malignancy based upon post-FNAB cytology (Bethesda V, VI), were confirmed by histological examination performed after thyroidectomy by two experienced pathologists as a routine medical procedure.

### Statistical analysis

All calculations were performed using Statistica 10 software by StatSoft. p value of less than 0.05 was considered statistically significant. The Mann–Whitney *U* test was used to compare age of the patients as well as diameter and SUV_max_ of the focal lesion. Spearman’s rank correlation coefficient was used to assess the correlation between size and SUV_max_ in thyroid lesions.

## Results

6614 PET/CT examinations performed in 5520 patients were analyzed. Diffused uptake of ^18^F-FDG was present in 109 patients (2.1%, 76 women and 33 men). Focal uptake was found in 122 patients (2.3%, 83 women and 39 men). Mean age of the patients with diffuse uptake was 60.7 years, in patients with focal uptake –61.0 years. No significant difference between genders in both groups was found (p>0.05).

Mean size of focal lesions was 14.8±9.0 mm (range 6–63 mm). Mean SUV_max_ was 4.3±4.1 in patients with focal uptake and 3.2±1.1 in patients with diffused uptake.

**Figure 3:**
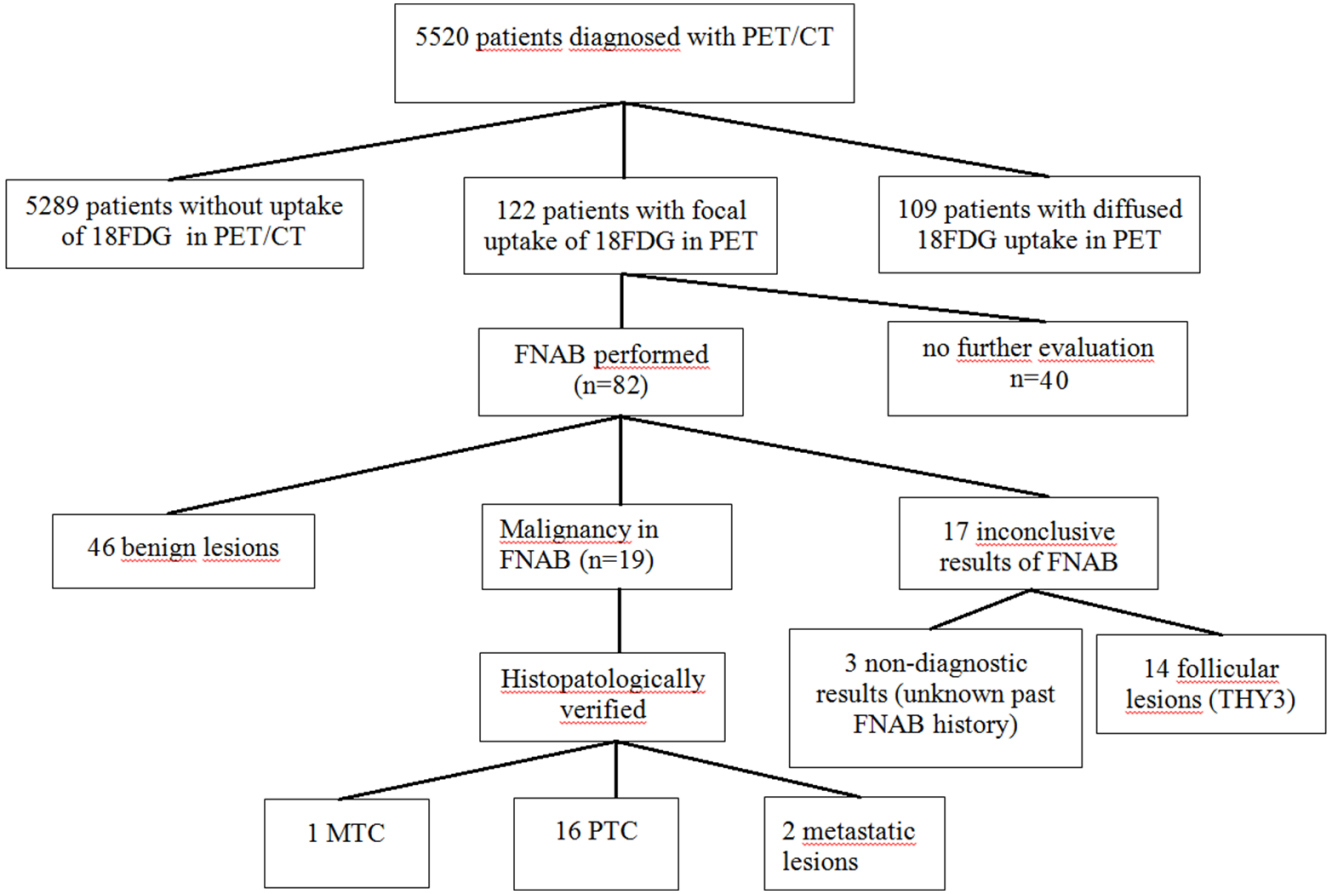
Diagram showing the protocol of the study and results of succeeding diagnostic steps.

Of the 122 patients with focal thyroid ^18^F-FDG activity, 82 patients (67.2%) underwent further thyroid evaluation using FNAB. The remaining 40 patients were not studied further due to severity of the malignant disease, overall poor general condition, necessity for rapid oncological treatment, or patient’s denial. Benign thyroid lesions were diagnosed in 46 patients (56.1%); histopathology was available in 3 cases. Malignant lesions were detected in 19 patients: 16 papillary thyroid carcinomas, one medullary thyroid carcinoma and two cases of metastases (one from laryngeal and one from esophageal cancer). Seventeen patients had inconclusive results of FNAB (3 non-diagnostic biopsies, 14 follicular lesions) and further results of repeated FNABs and/or histopathology were unavailable. All of the subjects were advised to repeat the biopsy in cases of follicular lesions of undetermined significance (Bethesda III) or undergo a surgical procedure in cases where there was suspicion of follicular neoplasm (Bethesda IV), but this data has yet to be analyzed. Study protocol and flow are presented in figure 3.

Mean SUV_max_ for malignant lesions was 7.1±8.2 (median 3.5). In the cases of benign lesions, mean SUV_max_ was 3.2±2.8 (median 2.4). The difference between the groups was statistically significant (p = 0.01). Comparison between benign and malignant nodules is shown in table 1. Mean SUV_max_ for DTC was 7.6±9.1 (median 3.3). In the 17 cases of lesions with inconclusive results of FNAB, mean SUV_max_ was 3.5 (SD 3.4, median 2.5) and was not significantly higher than in cases of benign lesions (p = 0.95). For follicular lesions (n = 14), mean SUV_max_ was 3.8±3.7 (median 2.6). This value also did not differ significantly from the SUV_max_ found in benign nodules (p = 0.77).

**Table 1:** Comparison between parameters estimated in PET/CT of benign and malignant nodules.

Feature	Benign lesions	Malignant lesions	p-value
Age	60.0±12.8	62.3±10.2	0.39
Mean size (mm) ± SD	14.1±6.2	18.0±14.1	0.64
SUV_max_ – range	1.4–17.5	1.8–33.6	
Mean SUV_max_ ± SD	3.2±2.8	7.1±8.2	0.01

No correlations between lesions diameter and SUV_max_ in the whole group of nodules (p = 0.16) was found. In cases of benign lesions there was also no significant correlation (p = 0.77). However, in the group of malignant lesions, a positive correlation between the lesion’s diameter and SUV_max_ was observed (p = 0.03, r = 0.57).

**Figure 4:**
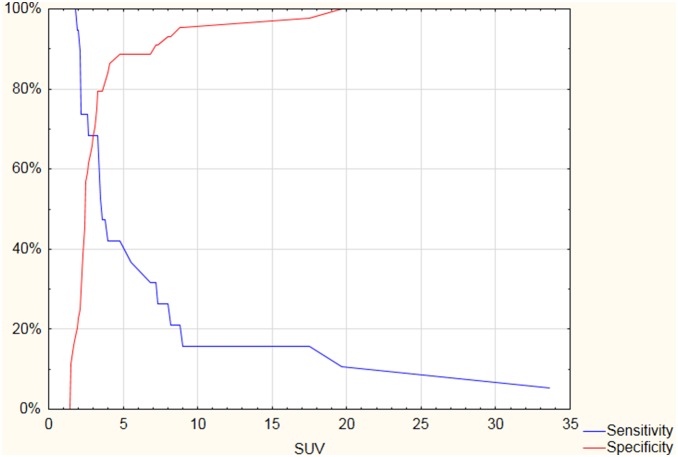
Graph showing sensitivity and specificity for particular SUV_max_ (standardized uptake value) thresholds in the prediction of malignant thyroid lesions.

**Figure 5:**
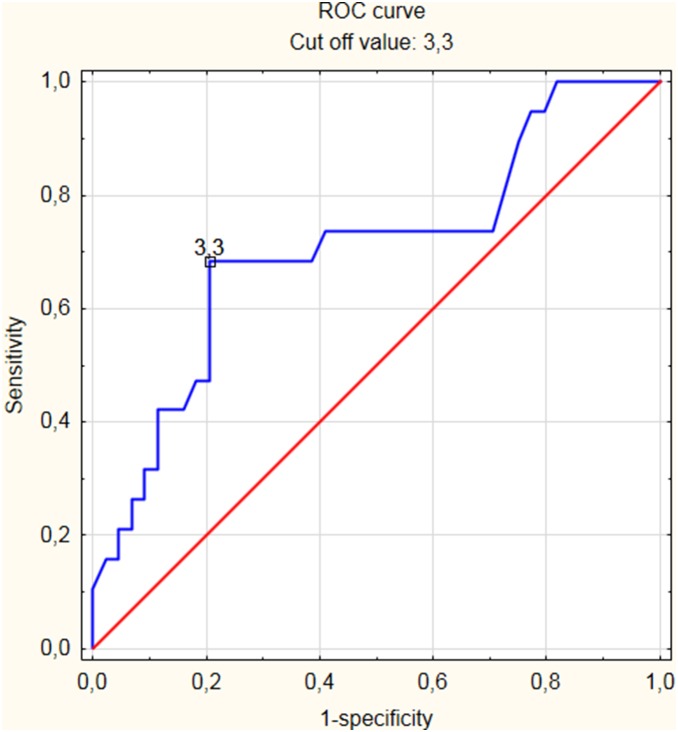
Receiver operating characteristic (ROC) curve showing the correlation between standardized uptake value (SUV_max_), sensitivity and specificity in the prediction of malignant thyroid lesions. Cut off value – 3.3.

Malignant thyroid nodules accounted for 29.2% of lesions with conclusive FNAB results. Sensitivity, specificity and positive predictive values of selected SUV_max_ thresholds are shown in table 2 and figure 4. The risk of malignancy was 16.7% for lesions with a SUV_max_ under 3, 43.8% for lesions with a SUV_max_ between 3 and 6, and 54.6% for lesions with a SUV_max_ over 6. According to the receiver operating characteristic (ROC) curve, the optimal sub-max cut-off value is 3.3 (Fig. 5).

**Table 2:** Sensitivity, specificity and positive predictive values of selected SUV_max_ thresholds for malignant lesions.

SUV_max_ threshold	Sensitivity	Specificity	PPV
2	89,5%	25,0%	34,0%
3	68,4%	70,5%	50,0%
4	42,1%	86,4%	57,1%
5	36,8%	88,6%	58,3%
7	31,6%	90,9%	60,0%
9	15,8%	97,7%	75,0%

## Discussion

PET/CT using ^18^F-FDG is widely used in the initial staging, assessment of treatment response and evaluation of potential recurrence of different malignancies [4], [17], [18]. A wider availability of this imaging technique leads to the increase of patient referrals for PET/CT scans. Standardized uptake value, estimated using ^18^F-FDG PET/CT, is the main parameter used to quantify glucose metabolism in the detected lesions. In general, the value is higher in malignant tissues [15]. Normal thyroid tissue usually does not accumulate ^18^F-FDG [4], [19]. Significantly higher ^18^F-FDG uptake with a diffuse pattern of accumulation was described in patients with chronic thyroiditis [20] and Graves’ disease [21]. The incidence and potential explanation of focal uptake of ^18^F-FDG within the thyroid gland have already been evaluated in some studies [3], [5]–[12], [11], [25]–[27]. The diagnostic role of SUV_max_ in thyroid incidentalomas is not fully elucidated, as the published data on the differential diagnosis of benign and malignant thyroid nodules were inconsistent.

In our clinical center, incidental focal uptake of ^18^F-FDG in the thyroid was observed in 122 patients (2.3%). This prevalence is in approximate concordance to those reported in other studies (1,2–4,3%) [5], [6], [7], [8], [9], [10], and results of recent meta-analysis on the topic of thyroid incidentalomas in PET examination (focal uptake in 1.6%) [22]. The risk of malignancy was estimated at 14% to 50% [5], [6], [7], [9], [11], [13], the meta-analysis performed by Soelberg et al. estimated this risk to be 34.8% [22]. In our study, approximately 30% of the incidentalomas appeared to be malignant in further evaluations (FNAB and post-operative histopathology). However, it is worth to remember, that out of 122 patients with focal ^18^F-FDG uptake, conclusive results of FNAB or histopathology were available only in 82 cases. Moreover, 17 patients had inconclusive FNAB, without definite distinction between benign and malignant lesions, and were not included in the final analysis of the risk of malignancy and comparison of SUV_max_ between benign and malignant lesions.

As differentiated thyroid cancer (DTC) is characterized by increased expression of glucose transporters (GLUT 1) [5], [6], [7], [9], the appearance of elevated focal uptake of ^18^F-FDG in the thyroid should give suspicion of malignant transformation. Numerous studies indicated that malignant lesions had significantly higher SUVs_max_ than benign ones [6], [13], [23], [24]. However, comparable number of reports found SUV_max_ to be useless in the prediction of the cytological and histopathological character of lesions [11], [25], [26], [27].

According to our results, there was a significant difference (p<0.05) between SUVs_max_ of benign and malignant nodules (mean 3.2 vs. 7.1) (tab.1). Moreover, higher values of SUV_max_ were associated with a higher risk of malignancy (e.g. lesions with a SUV_max_ below 3.0 had the risk of malignancy lower than 20%, whereas in cases of lesions with a SUV_max_ above 5.0, the risk was close to 60%). Three out of 4 lesions with a SUV_max_ above 9.0 were malignant.

As mentioned above, three lesions with non-diagnostic FNAB results and 14 lesions with cytological results belonging to category III and IV in Bethesda classification (III- atypia of undetermined significance/follicular lesions of undetermined significance, IV – follicular neoplasm or suspicion of a follicular neoplasm) were not taken into account during the final analysis. These follicular nodules had mean SUV_max_ insignificantly higher than lesions definitely diagnosed as benign.

Additionally, three out of 19 malignancies were not differentiated thyroid cancers (DTC), as there was one medullary thyroid carcinoma and two cases of metastases – one from laryngeal and one from esophageal cancer. However, the SUVs_max_ calculated solely for DTCs were similar to those calculated for all malignancies. According to some reports, PET with the use of ^18^F-FDG may be useful in the assessment of aggressive MTC [27], [28], [29] and detection of laryngeal and esophageal carcinomas recurrence [30], [31], [32], which seems to explain our findings.

In our study, significant correlation between maximal diameter of the lesion and SUV_max_ was found in the malignancy group. No significant correlation was found in the group of benign lesions. These results suggest that PET/CT can be less effective in the evaluation of small thyroid cancers (such as microcarcinomas), as they have significantly lower SUVs_max_ than the larger ones. Similar correlations were already reported by Kim et al. [11], while others [33] did not obtain identical conclusions.

It can be concluded that subjects with incidental focal uptake of ^18^F-FDG are at the high risk of thyroid malignancy. A high value of SUV_max_ further increases the risk of malignancy and should indicate the necessity for further cytological or histological evaluation. However, as SUV_max_ correlated with the diameter of malignant lesions, small lesions with focal uptake of ^18^F-FDG should be interpreted cautiously.
